# Nesting Site and Plumage Color Are the Main Traits Associated with Bird Species Presence in Urban Areas

**DOI:** 10.3390/ani12091148

**Published:** 2022-04-29

**Authors:** Lucas M. Leveau, Isis Ibáñez

**Affiliations:** Departamento de Ecología, Genética y Evolución, Facultad de Ciencias Exactas y Naturales, Universidad de Buenos Aires—IEGEBA (CONICET—UBA), Ciudad Universitaria, Pab 2, Piso 4, Buenos Aires 1426, Argentina; isisagostina97@gmail.com

**Keywords:** avian, functional traits, Latin America, ordination analysis, phylogenetics, urban ecology

## Abstract

**Simple Summary:**

Urban areas are expected to grow in the next decades, filtering bird species from the regional pool based on their life history traits. The objective of this study is to determine different bird species responses to urbanization using ordination analysis, and to characterize their life history traits combining information about diet, habitat and plumage color. Species identified as ‘urban exploiters’ tended to nest in buildings and with uniform plumage, whereas those identified as ‘urban avoiders’ tended to be ground nesting species with variable plumage. A third type, ‘urban adapters’, tended to be tree-nesting species with a low diet breadth, intermediate plumage lightness, low presence of plumage sexual dimorphism and high presence of iridescence. The results suggest that nest predation and habitat loss may exclude ground nesting birds from urban areas. The high density of pedestrians in urban centers may favor uniform plumages in birds that enhance camouflage.

**Abstract:**

Urban areas are expected to grow in the next decades, filtering bird species from the regional pool based on their life history traits. Although the impact of urbanization on traits such as diet, habitat and migratory behavior has been analyzed, their joint role with other traits related to plumage color has not yet been analyzed. Urban characteristics such as impervious surfaces, human presence and pollutants may be related to dark and uniform plumages. The objective of this study is to determine different bird species responses to urbanization using ordination analysis, and to characterize their life history traits combining information about diet, habitat and plumage color. Birds were surveyed along urban–rural gradients located in three cities of central Argentina. Species associations with urban characteristics were assessed through principal component analysis. Two axes were obtained: the first related positively to urban exploiters and negatively to urban avoiders, and a second axis related negatively to urban adapters. The scores of each axis were related to species traits through phylogenetic generalized least squares models. Species identified as ‘urban exploiters’ tended to nest in buildings and have uniform plumage, whereas those identified as ‘urban avoiders’ tended to be ground-nesting species with variable plumage. A third type, ‘urban adapters’, tended to be tree-nesting species with a low diet breadth, intermediate plumage lightness, low presence of plumage sexual dimorphism and high presence of iridescence. The results suggest that nest predation and habitat loss may exclude ground nesting birds from urban areas. The high density of pedestrians and domestic animals, such as cats and dogs, in urban centers may favor uniform plumages in birds that enhance camouflage.

## 1. Introduction

Urbanization is expected to increase globally [1], impacting negatively on bird communities through habitat loss and fragmentation, pollution, and species invasions [2,3,4,5]. Several studies have shown that bird species composition in urban areas are molded by a filtering from the regional species pool surrounding the city [6,7,8,9,10,11]. 

The urban filter imposed on birds is linked to many bird life-history traits [12]. Bird species with varied diets and habitat use have been associated with urban areas [13,14,15,16,17,18,19]. Nesting site has also been strongly associated with bird responses to urbanization. Ground or shrub nesting bird species were negatively related to urban areas [6,16,18,20,21,22,23,24,25], whereas species that nest in buildings or rocks were favored by urbanization [21,25,26,27]. Migrant species have been negatively associated with urban areas [21,26,28], but see [18]. Gregarious foraging behavior has been positively associated with urban bird species [18,26,28]. Species with large body mass and clutch sizes seem to be favored by urbanization [6,18,29], but see [24,30]. 

Recently, plumage color was proposed as another life history trait associated with bird species presence in urban areas [29,31]. Bird color diversity was lower than expected by chance in urban centers of central Argentina, suggesting that plumage color was filtered by urbanization, favoring species with grey plumage [29]. Grey plumage may be favored in highly urbanized areas due to presence of the impervious surfaces of roads and buildings. In addition, sexual plumage dimorphism has been negatively related to bird species presence in urban areas [6,31], but see [26]. This lack of sexual dimorphism in urban areas could be related to more energy being invested in adaptations to survive in new environments. Therefore, birds could be avoiding predation or fighting infections, instead of allocating energy to sexual selection [6,31]. However, the joint role of plumage color and other traits such as diet, habitat or residency status leading to bird presence in urban areas has not been analyzed yet. This information is fundamental to understand and predict how urbanization will affect bird communities.

The relationship between bird plumage color and environmental gradients has been analyzed by measuring plumage lightness [32,33,34], which varies between light and dark. At large scales, dark plumages are predominant in humid regions and with high tree cover [33,34], which probably favor camouflage and parasite resistance. However, the impact of land use change on plumage lightness has not been assessed yet. Highly urbanized areas dominated by impervious surfaces could favor melanic bird plumages, which may improve camouflage and parasites resistance [35]. In addition, the analysis of intraspecific variation of plumage lightness could bring new insights about urbanization effects on bird species. Intraspecific plumage variation of lightness, such as the presence of bright plumage patches, may indicate both intra and interspecific communication [36,37]. In contrast, uniform plumage lightness may favor camouflage. For example, Stevens et al. [38] found that ground nesting birds with the lowest plumage color contrast with their background habitat had increased survival. In cities, uniformly grey plumage might help camouflage on asphalted impervious surfaces, whereas uniformly green plumage may favor camouflage on vegetation. Camouflage in urban environments is relevant due to the high pedestrian traffic, whose presence may be analogous to predation risk to birds [39,40]. The presence of dogs and cats in highly urbanized areas may also increase the perceived predation risk by birds [41]. Nevertheless, the relationship between intraspecific plumage variation and urbanization has not been analyzed yet.

Most studies analyzing which traits were related to species presence in urban areas have used the classification of Blair [42], which grouped species according to three categories: (1) urban exploiters, which have their maximum densities in highly urbanized areas; (2) urban adapters, which have their maximum densities in moderately urbanized areas; and (3) urban avoiders, which only thrive in rural or natural areas. Several authors have compared bird traits in urban exploiters/adapter versus urban avoiders [6,14,16,19,22], urban exploiters versus urban adapters [26] or urban versus rural species [43]. Other studies ordered species in a continuous dimension based on their densities in urban areas and the ratio between urban and rural densities [15] or the median radiance, a proxy of artificial light at night, occupied by each species [18]. The present study proposes to ordinate species in a multivariate space based on multiple urbanization measures, such as impervious cover composed of asphalted roads and buildings, habitat diversity and primary productivity. The use of these environmental variables could help to analyze simultaneously life history traits associated with exploiters, adapters and avoiders.

The objective of this study was to determine different species responses to urbanization in the Pampean region of central Argentina using ordination analysis. In addition, species responses to urbanization were related to their life history traits combining information about diet, habitat and plumage color. Urban exploiters are expected to be gregarious species, nesting in buildings, with large clutch sizes and broad diet and habitat use. Moreover, they are also expected to have dark, uniform plumage and an absence of plumage sexual dimorphism. Urban adapter species are expected to nest in trees [28], due to the tree availability in moderately urbanized areas. Urban avoiders are expected to nest on the ground due to the open habitats in rural areas, which have patches of grasslands that provide habitat for ground-nesting birds [28]. Finally, the open habitat of rural areas may favor light plumage that increases intraspecific communication in birds [44].

## 2. Material and Methods

### 2.1. Study Area

The research was conducted in three cities located in central Argentina: Mar del Plata (38°00′ S, 57°33′ W; 38 m.a.s.l.; 615,350 inhabitants), Balcarce (37°50′ S, 58°15′ W; 112 m.a.s.l.; 38,823 inhabitants), and Miramar (38°16′ S, 57°50′ W; 17 m.a.s.l.; 29,629 inhabitants, 2010 National census) (see [45]). The three cities are surrounded by the Austral Pampas, consisting mainly of grazing land, cropland, and exotic tree plantations. The climate is temperate, with a mean annual precipitation of 923.6 mm and a mean annual temperature of 14 °C (data from the National Meteorological Service, www.smn.org.ar, accessed on 15 January 2019). Because the maximum distance between the cities was 59 km, effects of latitude or climate were deemed negligible.

### 2.2. Bird Surveys

Bird surveys were conducted in three habitat types: (1) urban centers; (2) suburban areas composed of detached houses with gardens; and (3) rural areas, composed of crops and pastures (see [45] for details). Five transects of 100 × 50 m separated by at least 200 m were surveyed in each habitat type and city, totaling 45 transects. Birds were surveyed by walking in a straight line mid-transect for three to five minutes and recording bird songs or sightings on both sides of the transects (25 m each). Two visits were made during the breeding season (austral spring-summer 2011–2012) and two visits during the breeding season 2012–2013, totaling four visits to each transect. Surveys were made during the first 4 h after dawn on days without rain or strong winds. All birds seen or heard that used the space within the transect for perching, walking or foraging were counted, except for those flying over the top of buildings or trees or below that height but without feeding activity.

### 2.3. Environmental Characteristics

Eight variables were measured in each transect: (1) impervious cover, (2) pedestrian traffic, (3) car traffic, (4) motorcycle traffic, (5) bicycle traffic, (6) minimum distance to rural areas, (7) habitat diversity and (8) primary productivity. Impervious cover and habitat diversity were measured visually by two 25 m radius circles, one in the center of the first 50 m along transects and the other in the center of the remaining 50 m (see [45] for more details). Impervious cover was characterized by the mean percent building and pavement cover of the two circles in each transect. Habitat diversity was calculated as the mean Shannon index of the two circles in each transect, incorporating the percent cover of trees, shrubs, lawn, herbaceous vegetation, cultivated land and buildings. Pedestrian, car, motorcycle and bicycle traffic were measured during three minutes simultaneously to bird surveys. Therefore, the mean values of the four visits were calculated for each transect. Minimum distance to rural areas was measured for each transect with Google Earth Pro. Finally, primary productivity was estimated using the Normalized Difference Vegetation Index (NDVI), which is a measure of greenness that correlates positively with net primary productivity [46,47] and is assumed to correlate positively with production of food available to birds [48]. NDVI for each transect was calculated as the mean value obtained of four images of the MOD13Q1 product [49], which correspond to the four visits to each transect (see [45] for more details).

### 2.4. Bird Species Urbanness

The species use of urban habitats, or species urbanness, was calculated by ordering bird species according to environmental characteristics of the transects they occupied along the urban–rural gradients. Therefore, the median value of the eight environmental characteristics was calculated for each species (see [18]). A matrix of bird species as rows and environmental characteristics as columns was used to perform a principal components analysis (PCA) with the rda function of the vegan package in R version 3.6.1 [50,51]. PCA reduces a matrix of variables in a few axes through linear combinations [52]. Only those axes with eigenvalues greater than 1 were considered [53]. Axes were characterized according to their relation to environmental variables. The principal component score values for each bird species were used as the urbanness score.

### 2.5. Life History Traits

Life history traits of species were characterized by eleven traits (Table S1): (1) diet breadth, (2) habitat breadth, (3) body mass (g), (4) clutch size, (5) migratory status, (6) nesting site, (7) gregariousness, (8) plumage mean lightness, (9) plumage lightness variation, (10) iridescent plumage presence, and (11) sexual plumage dimorphism presence. 

Diet breadth, habitat breadth and body mass were obtained from the data provided in the Elton Traits 1.0 database [54], which contains the percentage use of different food and foraging substrates by each species. Food items included invertebrates, endothermic vertebrates, ectothermic vertebrates, fish, carrion, fruit, nectar, seed and other plant material. Foraging substrate included below water surface, around water surface, ground, understorey, medium-high stratum, canopy and air. Diet and habitat breadth were calculated using Rao’s quadratic entropy B_D_ [19,55] using the nichevar function of the indicspecies package [56]. The Rao’s index varies between 0, indicating the use of only one food item or substrate, and 1, indicating the highest variety of food items or substrates. Clutch size was obtained from the Handbook of the Birds of the World (HBW) online (https://www.hbw.com/, accessed on 15 January 2019). The residency status of species was classified as resident or migratory, based on Narosky and Di Giacomo [57]. Nesting site was classified as ground or shrub, tree, and building based on de la Peña [58]. Brood parasites were included in tree nesting because the two parasite species, the Shiny Cowbird (*Molothrus bonariensis*) and the Screaming Cowbird (*Molothrus rufoaxillaris*), use mainly host species that nest in trees in the study area (L.M. Leveau, pers. Obs.). Gregariousness was considered as foraging or roosting in groups and based on de la Peña [59] and personal observations.

Plumage lightness and the variation of plumage lightness were quantified using Red-Green-Blue (RGB) values obtained from plates of the HBW online database (https://www.hbw.com/, accessed on 7 May 2020) (see also [27]). Plates were captured in .png archives, and opened in ImageJ [60]. Then, lightness was computed as (R + G + B)/3, which varies between 0 (pure black) and 255 (pure white). Lightness values were obtained from the head/crown, throat, breast, belly, coverts, primaries, nape/back, and tail in a similar way to Dale et al. [27]. Therefore, these values were averaged to obtain plumage lightness for each species. The plumage lightness variation was calculated as the coefficient of variation of lightness for all the plumage patches. In the case of sexual plumage dimorphic species, only male plumage was measured. Although males in sexual dimorphic species are darker than females, there is a positive correlation of plumage lightness between sexes within species [33]. Presence of iridescent plumage and sexual dimorphism were obtained from the Aves Argentinas cellphone app [61].

### 2.6. Statistical Analysis

The response to urbanization might be influenced by phylogenetic relatedness between species. Therefore, a phylogenetic generalized least squares model (PGLS) was performed to relate our response variables, the PCA species scores, with the predictor life history traits, using the gls function of the nlme package [62]. A dated phylogeny of all species in this study was created using the BirdTree database [63] (Jetz et al. 2014) and incorporated into the analysis. A total of 1000 phylogenies were downloaded from the Ericsson backbone phylogeny [64]. A 50% majority-rule consensus tree was constructed using the software TreeAnnotator [65]. The phylogeny was added as a correlation structure with a Brownian motion process of evolution on the tree, using the corBrownian function of the ape package [66]. The tree was used in R with the functions read.nexus and as.phylo of the ape package [66].

Model selection was performed through a backward elimination of non-significant variables (*p* > 0.05) from the whole model that included all predictors. A quadratic relationship was included in the model to explore non-linear relationships between plumage lightness and bird species urbanness. The non-significant variables were excluded using Likelihood ratio tests (LRT) (*p* > 0.05). Pseudo-R2 of models were calculated using the McFadden [67] formula: Pseudo-R2 = 1 − (Residual deviance/Null Deviance). Models were plotted using the visreg package [68].

## 3. Results

**Figure 1:**
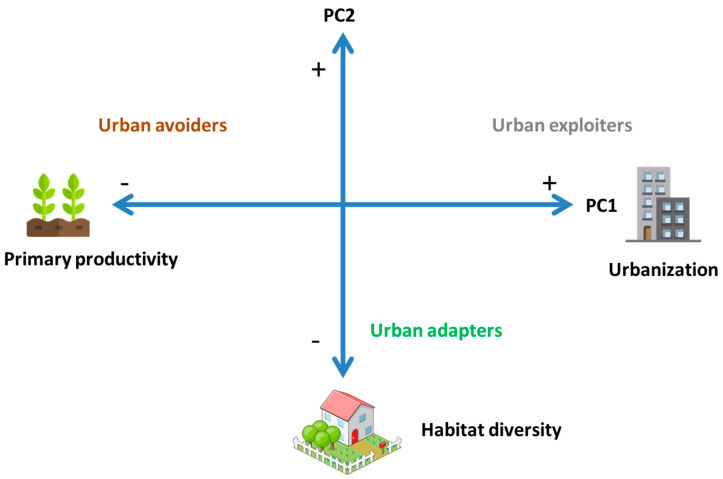
Schematic representation of the ordination of bird species and environmental variables. Urbanization includes the median impervious cover, vehicle and pedestrian traffic, and distance to rural areas for the records of each species. Primary productivity is the median NDVI value for the records of each species. Habitat diversity is the median Shannon index calculated with percent cover of vegetation strata and building cover.

A total of 54 species were analyzed (Table S1). The ordination analysis showed two main axes of bird species urbanness (Table 1, Figure 1 and Figure S1). The first principal component (PC1) was positively related to urban conditions, such as impervious cover, vehicle and pedestrian traffic and distance to rural areas (Table 1). Bird species positively related to PC1 were known urban exploiters, such as the Rock Dove (*Columba livia*), the Eared Dove (*Zenaida auriculata*) and the House Sparrow (*Passer domesticus*) (Figure 1 and Figure S1, Table S2). In contrast, the PC1 was negatively related to NDVI, a proxy of primary productivity, and to urban avoiders such as the Rufous-collared Sparrow (*Zonotrichia capensis*) and the Grassland Yellow-Finch (*Sicalis luteola*). The second axis (PC2) was negatively related to habitat diversity, and therefore, urban adapter species such as the White-throated Hummingbird (*Leucochloris albicollis*) and the Small-billed Elaenia (*Elaenia parvirostris*) tended to have negative scores (Table 1 and Table S2, Figure 1 and Figure S1).

**Figure 2:**
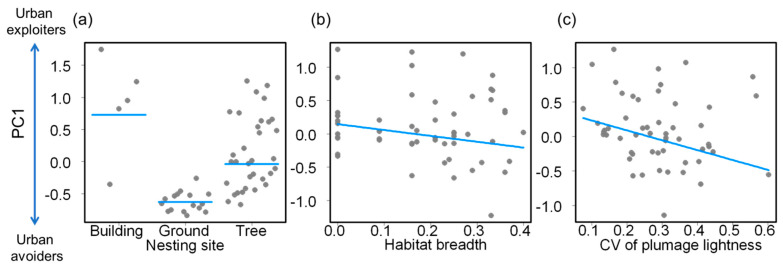
Relationship between the first principal component score values (PC1) of bird species and (**a**) nesting site, (**b**) habitat breadth, and (**c**) the intraspecific coefficient of variation (CV) of plumage lightness. Positive PC1 score values are related to urban exploiters, whereas negative values are related to urban avoiders. In (**a**) lines indicate mean values, whereas in (**b**,**c**) the lines indicate the fit of the phylogenetic generalized least square model.

The final model of PC1 score values included nesting site, habitat breadth and plumage lightness variation (LRT = 26.57, *p* < 0.001, pseudo-r^2^ = 0.23; Table 2). Positive scores were related to species that nest in buildings, whereas negative scores were related to species that nest on the ground and on trees (Figure 2a). Therefore, urban exploiters mainly nested on buildings, whereas urban avoiders nested on the ground. PC1 species scores tended to decrease with increasing habitat breadth (Figure 2b). Thus, urban exploiters used a lower variety of vegetation strata than urban avoiders. Plumage lightness variation increased with decreasing PC1 score values (Figure 2c). Therefore, urban exploiters had uniform plumage lightness, whereas urban avoiders had more variable plumage lightness. 

**Figure 3:**
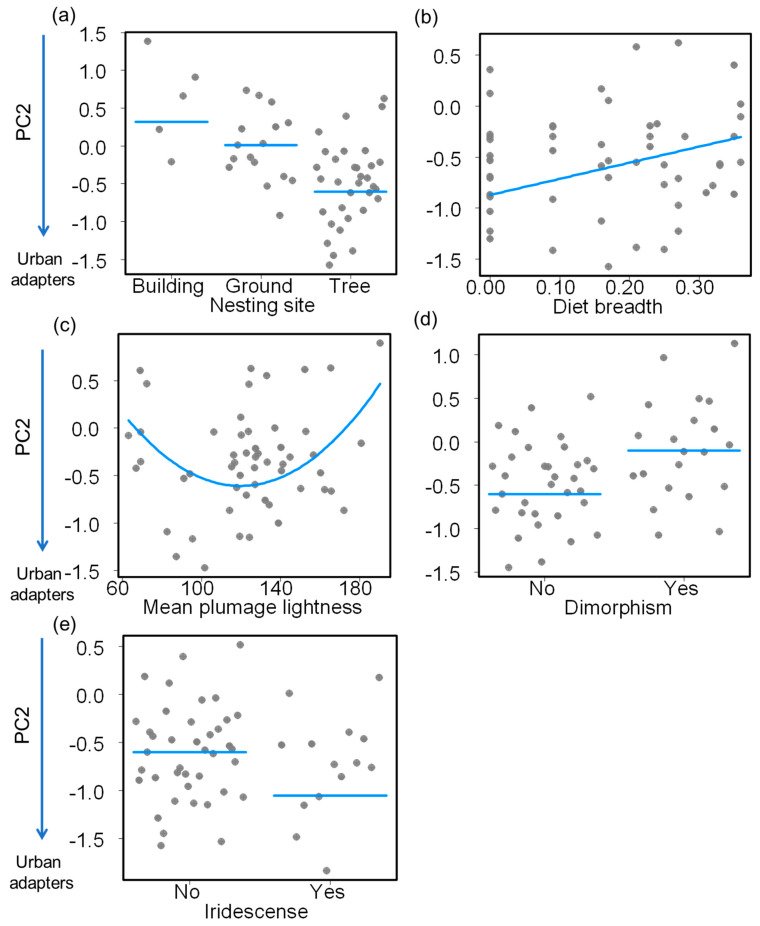
Relationship between the second principal component score values (PC2) of bird species and (**a**) nesting site, (**b**) diet breadth, (**c**) mean plumage lightness, (**d**) plumage dimorphism presence and (**e**) the presence of plumage iridescence. Negative PC2 score values are related to urban adapters. In (**a**,**d**,**e**) lines indicate mean values, whereas in (**b**,**c**) lines indicate the fit of the phylogenetic generalized least square model.

The final model for PC2 species score values included nesting site, diet breadth, plumage lightness, dimorphism presence and iridescence presence (LRT = 43.25, *p* < 0.001, pseudo-r^2^ = 0.31; Table 2). Negative scores were related to species that nest in trees (Figure 3a). Therefore, urban adapters were associated with species that nest in trees. Positive PC2 score values were associated with species that nest in buildings and on the ground and corresponding to urban exploiters and avoiders (Figure 3a). PC2 scores increased with diet breadth, indicating that urban adapters had low diet breadth (Figure 3b). Negative PC2 score values had intermediate plumage lightness, whereas positive PC2 score values had both the lowest and the highest plumage lightness (Figure 3c). Therefore, urban adapters had intermediate plumage lightness, whereas urban exploiters and avoiders had both dark and bright plumages. Negative PC2 values were associated with the absence of conspicuous plumage sexual dimorphism and the presence of plumage iridescence (Figure 3d,e). Thus, urban adapters had a lower plumage sexual dimorphism but a higher iridescence than exploiters and avoiders.

## 4. Discussion

The results obtained showed that nesting site, diet, habitat use, and plumage characteristics are associated with different bird responses to urbanization. The use of ordination analysis allowed obtaining three types of bird responses to urbanization, which coincided with the exploiter-adapter-avoider classification [42]. Urban avoider species were characterized by nesting on the ground and with variable plumage lightness, whereas urban exploiters were characterized by nesting on buildings and uniform plumage colors. Urban adapters were characterized by nesting in trees, specialized diets, intermediate plumage lightness, low presence of sexual plumage dimorphism and tended to have a high presence of plumage iridescence. 

Nesting site seems to be the main driver of species responses to urbanization, and ground nesting species were most negatively related to urbanization. This result agrees with the majority of studies analyzing avian species responses to urbanization [12]. Bird species nesting on the ground may suffer from habitat loss in urban areas, as they need natural herbaceous vegetation to nest [58], and this type of vegetation is replaced by lawn [45]. Nest predation and human disturbance in urban areas can also negatively affect ground nesting birds [69,70]. In contrast, bird species that nest in buildings or rocks are obviously favored by the availability of nesting sites in urban areas. Intermediate levels of urbanization consisting of yards with trees may be especially favorable for urban adapter species that nest in trees.

Habitat breadth tended to be lower in urban exploiters than in urban avoiders. This result is in line with the foraging behavior of several urban exploiters, such as the Rock Dove or the House Sparrow, which mostly search food on the ground. 

Urban exploiters were characterized by uniform plumage lightness, whereas avoiders had the highest plumage lightness variation. Low plumage lightness variation in urban exploiter species may favor species camouflage [29], as species with uniform plumage color can be less easily detected by humans or other predators [38,71]. Conversely, plumage lightness variation in urban avoiders may favor intra and interspecific communication by increasing conspicuousness [72,73,74]. In this study, urban avoider species such as the Fork-tailed Flycatcher (*Tyrannus savana*) and the Double-collared Seedeater (*Sporophila caerulescens*) had dark plumage in their dorsal parts, which probably enhances camouflage for protection from predators attacking from above or behind [38], whereas their light ventral plumage can enhance conspicuous signaling [73]. 

Urban adapters were related to diet specialization. Several urban adapter species, such as the White-throated Hummingbird and the Small-billed Elaenia specialize on one food type, nectar and invertebrates, respectively [54]. The results obtained contrast with other analyses made at global scale [19] and in Australia [18], which found a higher diet breadth in urban exploiters and adapters than in urban avoiders. Differences between studies may be related to the method used to classify bird species responses to urbanization. Palacio [19] used a binary exploiter/avoider classification, whereas Callaghan et al. [18] used a continuous index of bird species urbanization. Conversely, this study used an ordination method that enabled the classification of species in two dimensions, one that characterizes the exploiter/avoider continuum, and another dimension that characterizes urban adapters.

Plumage sexual dimorphism was negatively related to urban adapter species. This result agrees with those found by Croci et al. [6] and Iglesias-Carrasco et al. [31]. These authors proposed that urban exploiter and adapter species invest more energy in adaptations to survive in new environments, such as avoiding predation or fighting infections, instead of allocating energy to plumage development and maintenance that are related to sexual selection. However, the results obtained in this study did not find a low plumage sexual dimorphism presence in urban exploiters. Therefore, species present in highly urbanized areas may still allocate energy to plumage development and maintenance related to sexual selection due to relaxed predation or abundant food resources.

On the other hand, the presence of iridescent plumage tended to be higher in urban adapters than in urban exploiters and avoiders. Iridescence has been associated with courtship displays [75], which can be performed in precise moments when bathed directly in sunlight [76]. These courtship displays avoid unnecessary exposure to predators in urban environments, such as cats and raptors.

Urban adapter species were associated with intermediate plumage lightness, whereas the opposed side of the ordination consisting of urban exploiters and avoiders had extreme values of plumage lightness. It is probable that the semi-open habitat structure of suburban areas favors intermediate plumage lightness for enhancing camouflage. On the other hand, light plumage in urban avoiders, such as the White-tailed Kite (*Elanus leucurus*), may favor light reflectance and communication in open areas [44,77]. Urban avoiders also had predominantly dark plumages, as in the case of the Spectacled Tyrant (*Hymenops perspicillata*) and the Yellow-winged Blackbird (*Agelasticus thilius*). However, these species had small patches with bright colors in their wings, which may have a function for intraspecific communication.

Unlike other studies [6,18,21,26,28,29], the results obtained failed to find significant relationships between migratory status, clutch size, body size, flocking behavior and bird species urbanness. These contrasting results may be related to several factors, such as the methods used to classify bird species urbanness, the use of bird abundance or presence/absence data, the statistical approach and the spatial scale of analysis. For example, Callaghan et al. [18] found that certain traits, such as diet generalism, changed their relationship with bird urbanness depending on whether phylogenetic modelling was used or not. In addition, the methods used to classify bird urbanness varied from dichotomic categories [6,19,23,31] to continuous indices of bird urbanness on a single axis of impervious cover [18]. This study used a continuous index that ordered bird responses in multiple axes, considering simultaneously exploiters, adapters and avoiders. Finally, Kinnunen et al. [78] found that city characteristics such as compactness and socioeconomics variables, not considered in the present study, can be related to bird life history traits.

## 5. Conclusions

This study analyzed the relationship between bird species urbanness and traits such as diet, habitat and plumage color. Nesting site was strongly associated with bird species urbanness, with bird species nesting preferentially in buildings in highly urbanized areas, whereas species present in suburban areas nested mostly in trees. In contrast, bird species in rural areas nested mainly on the ground. Bird species present in highly urbanized areas had a uniform plumage color, suggesting a role of camouflage with the impervious surfaces. In contrast, bird species in non-urban areas had more contrast in their plumage patches, suggesting a role in intra and interspecific communication. The results obtained highlight the importance of considering plumage color when analyzing urbanization filters on bird species. Moreover, this analysis used an ordination method to classify species responses to urbanization, allowing characterization of traits of exploiters, adapters and avoiders. However, this study did not consider several traits, such as brain size, feeding innovation or song frequency, which has been associated with bird species responses to urbanization [16,23,43,79]. Therefore, future analyses that incorporate plumage color, song frequency and feeding innovations are necessary to obtain a more complete idea of how urbanization filters bird species.

## Figures and Tables

**Table 1:** Results of the principal component analysis (PCA) between species and their median values of the environmental variables. Numbers are loadings between each environmental variable and the first and second principal components (PC1 and PC2, respectively).

Environmental Variables	PC1	PC2
Impervious cover (%)	0.95	−0.15
Primary productivity (NDVI)	−0.94	0.06
Habitat diversity (H index)	0.23	−0.95
Car traffic	0.75	0.25
Pedestrian traffic	0.87	0.19
Bicycle traffic	0.84	0.07
Motorcycle traffic	0.73	0.14
Minimum distance to rural areas (m)	0.86	−0.11
Eigenvalues	5.12	1.07
Proportion of variance explained	0.64	0.13

**Table 2:** Final phylogenetic generalized least square models showing the relationship between (a) the first principal component species scores (PC1), (b) the PC2 and life history traits along urban-rural gradients of central Argentina. Nest in building, dimorphism absence and iridescence absence are included in the intercept.

	Value	Std. Error	t-Value	*p*-Value
(a) PC1				
Intercept	1.325	0.614	2.158	0.036
Nest—ground	−1.359	0.303	−4.490	<0.001
Nest—tree	−0.765	0.265	−2.888	0.006
Habitat breadth	−0.880	0.460	−1.914	0.062
CV of plumage lightness	−1.429	0.490	−2.916	0.005
(b) PC2				
Intercept	3.129	1.192	2.625	0.012
Nest—ground	−0.307	0.414	−0.740	0.463
Nest—tree	−0.921	0.370	−2.488	0.017
Diet breadth	1.579	0.692	2.282	0.027
Mean plumage lightness	−0.052	0.016	−3.212	0.002
Mean plumage lightness^2^	<0.001	<0.001	2.935	0.005
Plumage dimorphism—presence	0.503	0.188	2.681	0.010
Iridescence—presence	−0.452	0.231	−1.954	0.057

## Data Availability

The datasets generated during and/or analysed during the current study are available upon request to the corresponding author.

## Supplementary Materials

The following supporting information can be downloaded at: https://www.mdpi.com/article/10.3390/ani12091148/s1, Figure S1: Biplot with the results of the principal component analysis showing the relationships between species and environmental variables. NDVI: Normalized Difference Vegetation Index, H: habitat diversity (Shannon index), Dist_rur: minimum distance to rural areas; Table S1: Life history traits of species observed along the urban-rural gradients of central Argentina. Light_mean: mean plumage lightness between plumage patches, Light_cv: coefficient of variation of plumage lightness between plumage patches, Diet_var: diet breadth, Habitat_var: habitat breadth, Dimorph: presence of sexual plumage dimorphism; Table S2: Species scores of the first and the second principal components analyzing the relationship between environmental variables of species. See more details in Methods.
