# Supplementary material for: Nesting Site and Plumage Color Are the Main Traits Associated with Bird Species Presence in Urban Areas
Source: Animals (Basel). 2022 Apr 29;12(9):1148. doi: 10.3390/ani12091148 (PMC9099748; doi:10.3390/ani12091148)
Supplement: Supplementary file 1 [file animals-12-01148-s001.zip › animals-1651113-supplementary.pdf]

Table S1. Life history traits of species observed along the urban-rural gradients of central Argentina. Light_mean: mean plumage lightness between plumage patches, Light_cv: coefficient of variation of plumage lightness between plumage patches, Diet_var: diet breadth, Habitat_var: habitat breadth, Dimorph: presence of sexual plumage dimorphism.

Species	Mass (g)	Resident	Nesting type	Light_mean	Light_cv	Clutch	Diet_var	Habitat_var	Dimorph	Iridescense	Flock
Agelaioides_badius	45.25	resident	tree	116.344375	0.21237977	6.5	0.24	0.31	no	no	yes
Agelasticus_thilius	31.5	resident	ground	69.22225	0.1163838	4	0.25	0.21	yes	no	yes
Anthus_correndera	20.9	resident	ground	132.297875	0.26596068	4	0.09	0.17	no	no	no
Anumbius_annumbi	41.5	resident	tree	165.414375	0.2436349	4	0.16	0.25	no	no	no
Asio_flammeus	322.61	resident	ground	126.982125	0.18054309	4	0	0.09	no	no	no
Athene_cunicularia	150.61	resident	ground	133.19075	0.13355342	5	0.23	0.17	no	no	no
Chloris_chloris	25.25	resident	tree	144.85	0.10036078	5	0	0.33	yes	no	no
Chlorostilbon_lucidus	3.5	migrant	tree	106.41625	0.07500978	2	0.09	0.09	yes	yes	no
Colaptes_campestris	158	resident	tree	95.402625	0.40992282	4.5	0.09	0	yes	no	no
Colaptes_melanochloros	127.27	resident	tree	87.370375	0.44473729	4	0.17	0.25	yes	no	no
Columba_livia	354.2	resident	building	124.073125	0.28967658	2	0.27	0.16	yes	yes	yes
Columbina_picui	47	resident	tree	162.416625	0.29283236	2	0	0	yes	yes	yes
Elaenia_parvirostris	13.8	migrant	tree	172.00425	0.29578197	2.5	0.21	0.3333333	no	no	no
Elanus_leucurus	346	resident	tree	190.403875	0.21716327	3	0.09	0.16	no	no	no
Embernagra_platensis	45.5	resident	ground	119.890625	0.24410903	4	0.35	0.28	no	no	no
Falco_sparverius	114.61	resident	tree	141.358125	0.31585482	4.5	0.28	0.29	yes	no	no
Furnarius_rufus	46.42	resident	tree	165.701625	0.29035413	3	0.16	0.21	no	no	no
Guira_guira	141	resident	tree	160.15625	0.35928472	10	0.33	0.23	no	no	yes
Hirundo_rustica	20	migrant	building	122.775625	0.3047232	5	0.17	0.33	no	yes	yes
Hymenops_perspicillatus	22.9	resident	ground	63.134375	0.43228608	3	0	0	yes	no	no
Leistes_loyca	113	resident	ground	101.789875	0.13545949	4	0.25	0.23	yes	no	yes
Leistes_superciliaris	45.32	resident	ground	67.014125	0.22566755	5	0.32	0.09	yes	no	yes
Leucochloris_albicollis	6.25	resident	tree	120.106375	0.55801967	2	0.09	0.27	no	yes	no

Machetornis_rixosa	29.6	resident	tree	152.2295	0.30969878	3.5	0	0	no	no	no
Mimus_saturninus	63.7	resident	tree	180.911	0.28768052	3.5	0	0	no	no	no
Molothrus_bonariensis	41.49	resident	parasite	94.08925	0.31053392	10	0.21	0	yes	yes	yes
Molothrus_rufoaxillaris	47.5	resident	parasite	82.580375	0.40381392	9.5	0.27	0.09	no	yes	yes
Myiopsitta_monachus	120	resident	tree	128.663	0.20745374	6	0.36	0.36	no	yes	yes
Passer_domesticus	26.51	resident	building	119.50675	0.43259873	3.5	0.27	0.25	yes	no	yes
Patagioenas_maculosa	347	resident	tree	119.71775	0.33752273	1.5	0.23	0.16	no	no	yes
Patagioenas_picazuro	279	resident	tree	115.39775	0.23123535	1	0.36	0.36	no	yes	yes
Phalcoboenus_chimango	296	resident	ground	150.18975	0.19691086	2.5	0.33	0	no	no	yes
Pipraeidea_bonariensis	36	resident	tree	140.35075	0.16802295	3	0.17	0.33	yes	no	yes
Pitangus_sulphuratus	62.85	resident	tree	134.23725	0.43391881	3.5	0.36	0.29	no	no	no
Plegadis_chihi	616.89	resident	ground	68.97575	0.14085096	3	0.17	0.16	no	yes	yes
Progne_chalybea	42.9	migrant	building	122.7875	0.33109092	3.5	0	0.4	yes	yes	yes
Progne_elegans	41.98	migrant	building	72.4575	0.21700788	4	0	0.21	yes	yes	yes
Progne_tapera	32	migrant	tree	137.13125	0.36545762	4	0	0.37	no	no	no
Pseudoleistes_virescens	79.93	resident	ground	69.296875	0.27170688	5	0.35	0	no	no	yes
Rhynchotus_rufescens	843.47	resident	ground	124.3615	0.31081419	12	0.35	0.16	no	no	no
Serpophaga_subcristata	6.6	resident	tree	126.726625	0.56766195	2	0	0.3333333	no	no	no
Setophaga_pitiayumi	6.82	resident	tree	140.170875	0.16225652	2	0.23	0	no	no	no
Sicalis_flaveola	16.89	resident	tree	117.7735	0.21609466	3	0	0.24	yes	no	no
Sicalis_luteola	15.9	resident	ground	123.82625	0.14589306	2.5	0	0.21	yes	no	yes
Spinus_magellanica	13.6	resident	tree	114.198375	0.41466713	3.5	0.27	0.3333333	yes	no	yes
Sporophila_caerulescens	9.73	migrant	ground	116.8425	0.41064211	3	0.16	0.25	yes	no	yes
Tachycineta_leucorrhoa	19	migrant	tree	152.814375	0.36651774	6	0	0.16	no	no	yes
Troglodytes_aedon	10.85	resident	tree	156.57825	0.30315014	6	0.16	0	no	no	no
Turdus_rufiventris	69.44	resident	tree	91.183875	0.28916409	4	0.25	0	no	no	no
Tyrannus_melancholicus	37.4	migrant	tree	127.3715	0.34738166	2.5	0	0.25	no	no	no
Tyrannus_savana	31.9	migrant	tree	132.894875	0.60286391	2.5	0.21	0.25	no	no	no

Vanellus_chilensis	327	resident	ground	127.391125	0.1324625	4	0.09	0.16	no	yes	yes
Zenaida_auriculata	110.2	resident	tree	125.124375	0.18557253	2	0	0.36	yes	yes	yes
Zonotrichia_capensis	20.31	resident	ground	138.830875	0.25694333	2.5	0.31	0	no	no	yes

Table S2. Species scores of the first and the second principal components analyzing the relationship between environmental variables of species. See more details in Methods.

Species	PC1	PC2
Agelaioides_badius	-0.32	-0.18
Agelasticus_thilius	-0.40	0.89
Anthus_correndera	-0.51	-0.27
Anumbius_annumbi	-0.59	0.61
Asio_flammeus	-0.51	-0.25
Athene_cunicularia	-0.49	0.35
Chloris_chloris	0.94	-0.07
Chlorostilbon_lucidus	0.51	-0.12
Colaptes_campestris	-0.50	-0.79
Colaptes_melanochloros	-0.26	-0.85
Columba_livia	1.79	1.59
Columbina_picui	-0.31	-0.87
Elaenia_parvirostris	0.64	-0.80
Elanus_leucurus	-0.52	0.77
Embernagra_platensis	-0.54	1.01
Falco_sparverius	-0.58	0.30
Furnarius_rufus	0.21	-0.68
Guira_guira	-0.54	-0.22
Hirundo_rustica	-0.48	-0.24
Hymenops_perspicillatus	-0.52	0.77
Leistes_loyca	-0.52	-0.23
Leistes_superciliaris	-0.46	0.93
Leucochloris_albicollis	0.82	-0.65
Machetornis_rixosa	0.13	0.35
Mimus_saturninus	-0.05	-0.43
Molothrus_bonariensis	0.17	-0.36
Molothrus_rufoaxillaris	-0.26	-1.39
Myiopsitta_monachus	-0.46	-0.42
Passer_domesticus	0.58	0.44
Patagioenas_maculosa	0.52	-0.40
Patagioenas_picazuro	0.40	-0.56
Phalcoboenus_chimango	-0.14	0.23
Pipraeidea_bonariensis	0.68	0.05
Pitangus_sulphuratus	0.36	-0.51
Plegadis_chihi	-0.52	0.77
Progne_chalybea	0.72	0.44
Progne_elegans	1.35	1.17
Progne_tapera	-0.52	-0.27
Pseudoleistes_virescens	-0.63	0.86
Rhynchotus_rufescens	-0.51	-0.25
Serpophaga_subcristata	0.48	-0.68
Setophaga_pitiayumi	1.45	-0.11

Sicalis_flaveola	-0.28	-0.39
Sicalis_luteola	-0.48	0.81
Spinus_magellanica	-0.29	-0.21
Sporophila_caerulescens	-0.47	0.74
Tachycineta_leucorrhoa	1.12	-0.30
Troglodytes_aedon	-0.02	-0.30
Turdus_rufiventris	0.84	-0.40
Tyrannus_melancholicus	-0.07	-0.48
Tyrannus_savana	-0.59	0.61
Vanellus_chilensis	-0.52	-0.27
Zenaida_auriculata	0.50	0.41
Zonotrichia_capensis	-0.28	-0.16

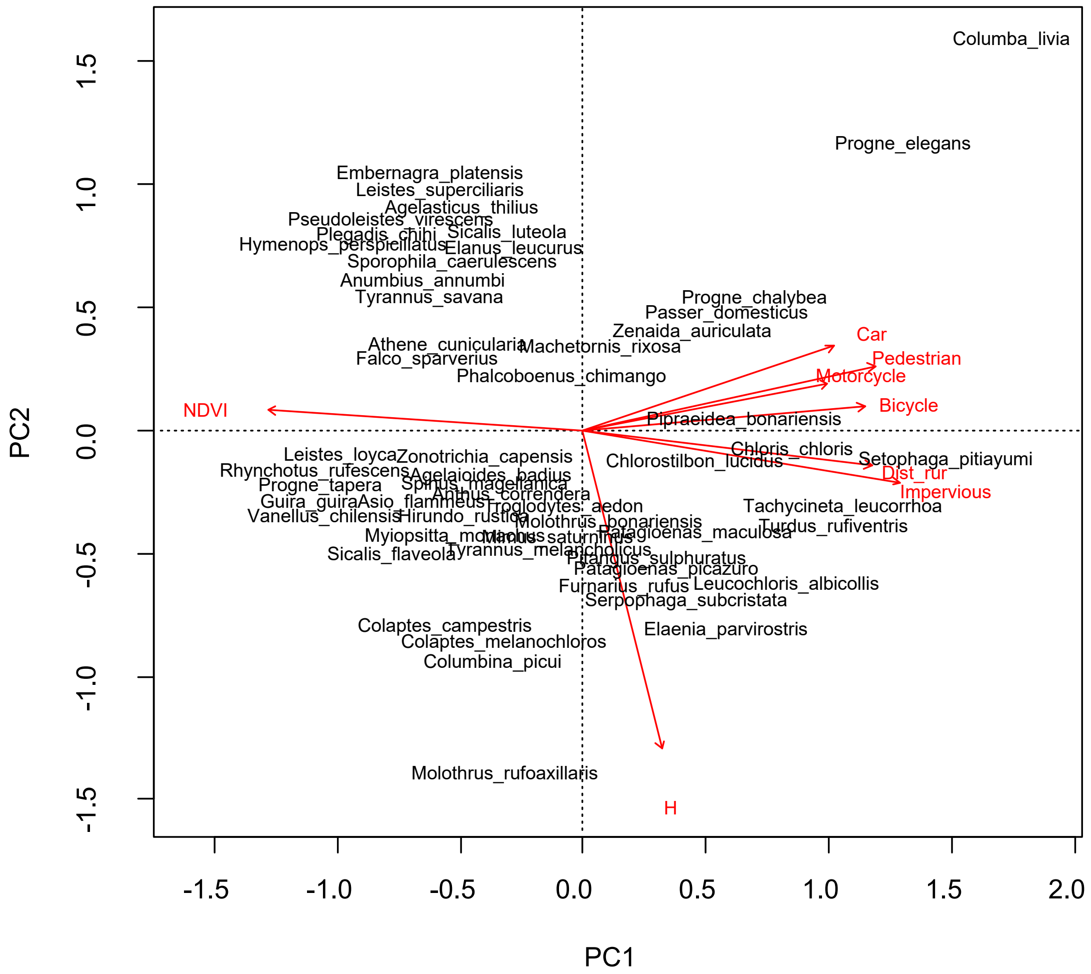

Figure S1. Biplot with the results of the principal component analysis showing the relationships between species and environmental variables. NDVI: Normalized Difference Vegetation Index, H: habitat diversity (Shannon index), Dist_rur: minimum distance to rural areas.
